# Influence of alumina air-abrasion for highly translucent partially stabilized zirconia on flexural strength, surface properties, and bond strength of resin cement

**DOI:** 10.1590/1678-7757-2019-0371

**Published:** 2020-01-31

**Authors:** Keiichi YOSHIDA

**Affiliations:** 1 Nagasaki University Hospital Clinic of Fixed Prosthodontics Nagasaki Japan Nagasaki University Hospital, Clinic of Fixed Prosthodontics, Nagasaki, Japan.

**Keywords:** Ceramics, Dental air abrasion, Dental bonding, X-ray crystallography, Flexural strength

## Abstract

**Objective:**

This study aims to evaluate the influence of different air-abrasion pressures and subsequent heat treatment on the flexural strength, surface roughness, and crystallographic phases of highly translucent partially stabilized zirconia (Y-PSZ), and on the tensile bond strength of resin cement to Y-PSZ.

**Methodology:**

Fully sintered zirconia specimens were ground with SiC paper (control) and/or air-abraded with 50 µm particles of alumina at 0.1, 0.15, 0.2, or 0.3 MPa or left as-sintered. After air-abrasion at 0.2 MPa (0.2AB), additional specimens were then heated to 1500°C, and held for one hour at this temperature (0.2AB+HT1h). Flexural strength and surface roughness were evaluated. Crystalline phase identification was also carried out using X-ray diffraction. Bonded zirconia specimens with self-adhesive resin cement were stored in distilled water at 37°C for 24 h, either with or without aging (thermal cycling 4-60°C/20000). Results were analyzed statistically by ANOVA and Tukey-Kramer tests.

**Results:**

The flexural strength decreased with the increase in air-abrasion pressure, while in contrast, the surface roughness increased. The lowest flexural strength and the highest roughness value were found for the 0.2AB and 0.3AB groups, respectively. All groups contained cubic-, tetragonal ( *t* )-, and rhombohedral ( *r* )-ZrO_2_ phases with the exception of the as-sintered group. Upon increasing the air-abrasion pressure, the relative amount of the *r* -ZrO_2_ phase increased, with a significant amount of *r* -ZrO2 phase being detected for the 0.2AB and 0.3AB groups. The 0.2AB+HT1h group exhibited a similar flexural strength and *t* -ZrO2 phase content as the as-sintered group. However, the 0.2AB group showed a significantly higher tensile bond strength (p<0.05) than the 0.2AB+HT1h group before and after aging.

**Conclusion:**

Micromechanical retention by alumina air-abrasion at 0.2 MPa, in combination with chemical bonding of a resin to highly translucent Y-PSZ using a MDP-containing resin cement may enable durable bonding.

## Introduction

All-ceramic dental restoration systems have become increasingly popular due to their good esthetics and biocompatibilities compared with metal-porcelain restorations. More specifically, zirconia ceramics have become one of the prime alternatives to metal-ceramic restorations due to their low cost compared with gold metal, in addition to the reduced laboratory costs for ceramic fabrication, and the ease of milling zirconia.^1^ Conventional zirconia ceramics are predominantly composed of fine tetragonal zirconia crystals containing 3 mol% yttria stabilizer, otherwise known as yttria-stablized tetragonal zirconia polycrystals (conventional Y-TZP). While being exceptionally strong, conventional Y-TZP ceramics tend to have a poor translucency. As zirconia and alumina have different refraction indices, the introduction of alumina can therefore decrease light transmission, due to which the 0.05 wt% alumina-containing Y-TZP is more translucent than its 0.25 wt% equivalent. The most recent strategy to improve the translucency of zirconia is to increase the significant cubic ( *c* ) phase in the zirconia structure.^2^ This has been achieved using a higher yttria content (4-6 mol%) to produce partially stabilized zirconia (Y-PSZ).^3^ This Y-PSZ is isotropic in different crystallographic direction, thereby decreasing the light scattering that occurs at grain boundaries, and rendering this material more translucent.^4 , 5^

The reliable bonding of zirconia ceramics has been reported by the mechanical retention and chemical bonding of resin luting cement to the ceramic substrate.^6^ Micromechanical retention can be achieved by increasing the surface area of the substrate with alumina air-abrasion. In addition, chemical and long-term durable bonding to zirconia ceramics has been demonstrated using phosphate monomer-containing resin cements^7 - 11^ or ceramic primers,^12 , 13^ such as 10-methacryloyloxy-decyl-dihydrogenphosphate (MDP). Furthermore, the alumina air-abrasion of conventional Y-TZP produces a protective surface compressive layer due to a tetragonal ( *t* ) to monoclinic ( *m* ) phase transformation. Air-abrasion can therefore either reduce^14 , 15^ or increase^16 - 19^ the flexural strength of conventional Y-TZP, depending on the type and size of alumina particles employed and the air pressure used, and the thermal history. No *m* -phase has been observed by heat treatment after air-abrasion with alumina in the conventional Y-TZP due to the *m→t* reverse phase transformation.^20^ Nevertheless, few studies have investigated the influence of air-abrasion with alumina and heat treatment on the flexural strength^21^ and surface characteristics including crystallographic phase^22^ of highly translucent Y-PSZ, and on the bond strength of resin cement to Y-PSZ.

Thus, the purpose of the present study was to examine the influence of air-abrasion with alumina at different pressures and subsequent heat treatment on the flexural strength, surface roughness, and crystallographic phases of highly translucent Y-PSZ. The influence of alumina air-abrasion and additional heat treatment on the bond strength of resin cement to highly translucent Y-PSZ was also evaluated. The null hypothesis tested in this study is that air-abrasion with alumina does not affect the flexural strength or surface characteristics of highly translucent Y-PSZ, nor does it influence the bond strength of resin cement to the treated Y-PSZ.

## Methodology

### Specimen preparation

CAD/CAM pre-sintered disks of highly translucent Y-PSZ (Aadva zirconia NT: 91 wt% ZrO_2_, 9 wt% Y_2_O_3_, trace Al_2_O_3_, 98.5 mm diameter, 14 mm thickness, GC Corp.; Tokyo, Japan) were used for this study. The disks were sectioned into bar specimens for measuring the flexural strength, and plate specimens for evaluating the surface characteristics and bond strength using a CAM machine (Aadva Mill LD-I, GC Corp.; Tokyo, Japan). All specimens were sintered in a sintering furnace (Austromat 664iSiC, Dekema GmbH; Freilassing, Germany) following the manufacturer’s recommendations. The furnace temperature was increased from room temperature to 1000°C at approximately 8°C/min, and then from 1000°C to 1500°C for 4.5 h, held at 1500°C for 2 h, decreased until 1000°C in one hour, cooled in the furnace until 200°C, and then cooled to room temperature. This surface condition was referred to as the as-sintered group. After sintering, specimens were ground with silicon carbide paper (#600, Struers; Ballerup, Denmark) under water-rinsing. The final dimensions of the specimens were 2.0 ± 0.2 × 2.0 ± 0.2 × 25.0 ± 0.5 mm^3^ for burs and 10 × 10 × 2 mm^3^ for plates. This surface condition was referred to as the control group.

All specimens were randomly assigned to eight groups including the as-sintered and control groups. Control specimens were then air-abraded with 50 µm particles of Al_2_O_3_ (Hi Aluminas, Shofu Inc.; Kyoto, Japan) at 0.1, 0.15, 0.2, or 0.3 MPa for 15 s at a distance of 10 mm (0.1AB, 0.15AB, 0.2AB, or 0.3AB) on one surface using a blasting machine (Basic classic II, Renfelt GmbH; Hilzingen, Germany). Subsequently, all specimens were cleaned ultrasonically in a distilled water-bath for 10 min and dried using oil-free air. Following air-abrasion with alumina at 0.2 MPa, additional specimens were then heated in the sintering furnace to 1500°C at a heating rate of 10°C/min, then held at 1500°C for 0 min (0.2AB+HT0) or 1 h (0.2AB+HT1h) prior to cooling to 200°C at a rate of 10°C/min, and then cooling to 20°C.

### Flexural strength

The flexural strength was determined using 3-point bending with bar specimens (n=10/group) according to ISO 6872, but without 45° edge chamfer. All specimens were loaded with the treated surface in tension. Tests were performed using a universal testing machine (AGS-10kNG, Shimazu Corp.; Kyoto, Japan) with a cross-head speed of 1.0 mm/min, and a span of 20.0 mm. The fracture load was recorded in N, and the flexural strength was calculated in MPa.

### Surface roughness analyses

The surface roughness values of three specimens from each group were measured using a laser scanning microscope (VK-X200, KEYENCE Co., Ltd; Osaka, Japan) equipped with a 50× objective. A laser beam with a spot size of 1 µm was used to scan the specimen surfaces, and this system exhibited a submicron resolution along all axes. Each surface was measured five times. The roughness values (R_a_) were obtained using the Microsoft Windows-based Match software package.

### X-ray diffraction analysis (XRD)

Following surface analyses, crystalline phase identification of the specimens (n=2/group) was carried out by X-ray diffraction (Empyrean, Malvern Panalytical; Almelo, Netherlands) using Cu-K_α_ radiation operating at 45 kV and 40 mA. Scans were performed over the 2θ range of 25-65° at a scan speed of 0.1347°/s. Quantitative phase analysis was carried out using the Rietveld refinement method by Highscore Plus software (Malvern Panalytical; Almelo, Netherlands).

### SEM evaluation

After XRD analyses of the crystalline phases, examination of the specimen microstructure was carried out using scanning electron microscopy (SEM, SU-70, Hitachi High-Technologies Corp.; Hitachinaka, Japan). Each specimen was sputter-coated with gold and analyzed at magnifications of 1000× and 5000×.

### Tensile bond strength

As in the case of the highly translucent Y-PSZ, sintered plate specimens (10 × 10 × 2 mm^3^) were also prepared using highly translucent Y-TZP (Aadva zirconia EI: 95 wt% ZrO_2_, 5 wt% Y_2_O_3_, trace Al_2_O_3_, GC Corp.; Tokyo, Japan) to compare the effect of alumina air-abrasion on the bond strength of resin cement to zirconia for the Y-TZP and Y-PSZ materials. The surface condition groups examined were the control and 0.2AB groups for Y-TZP, and the control, 0.2AB, and 0.2AB+HT1h groups for Y-PSZ.

Pieces of a polyethylene adhesive tape (approximately 50 µm thick), each containing a 4.0-mm-diameter circular hole, were affixed to the zirconia plate surface to define the bonding area. Stainless steel rods (SUS 304, 74Fe-18Cr-8Ni, 8 mm diameter, 12 mm height, Furuuchi Chemical Co., Ltd; Tokyo, Japan) were air-abraded with 50 µm particles of Al_2_O_3_ at 0.4 MPa for 15 s at a distance of 10 mm on one surface, then dried using oil-free air for 5 s. The zirconia plate specimens were then bonded to the stainless steel rods using resin cement (G-CEM ONE, GC Corp.; Tokyo, Japan). The resin cement was mixed and then applied in accordance with the manufacturer’s instructions. Excess cement was removed from the bonding margin using small disposable brushes. Light irradiation was performed by placing the tip of the light-emitting diode unit (power density of 1000 mW/cm^2^; Pencure; J. Morita Mfg. Corp.; Kyoto, Japan) on the opposite surface to the adhesive surface of zirconia for 40 s. The bonded specimens were then left undisturbed for 30 min at 20°C.

Sixteen specimens from each group were tested across two storage conditions (n=8/subgroup). Both subgroups were stored in 37°C distilled water for 24 h (TC0); thereafter, one subgroup was subjected to 20,000 thermal cycles (TC20000) between water baths (Rika-Kogyo; Hachioji, Japan) maintained at 4 and 60°C, with a dwelling time in each bath of 1 min *per* cycle.

The tensile bond strength was measured using a universal testing machine. The load was applied at a crosshead speed of 0.5 mm/min with the bonding surface parallel to the loading direction. The tensile bond strength was calculated by dividing the force at which bond failure occurred by the bonding area. The fractured interfaces of the debonded zirconia specimens were examined using an optical microscope (SMZ-10, Nikon Corp., Tokyo, Japan) at a magnification of 50× to classify the type of failure taking place during testing. Failure was classified as cohesive when the resin cement fractured, adhesive when the fracture occurred between the zirconia and the resin cement, and mixed when a combination of adhesive and cohesive failure occurred.

### Statistical analyses

The flexural strength and surface roughness results were analyzed by one-way analyses of variance (ANOVA) and the tensile bond strength results were analyzed by two-way ANOVA, followed by the Tukey-Kramer *post hoc* tests to compare the effects of each surface treatment or storage condition. The overall significance level was set at α=0.05. All statistical analyses were performed using BellCurve for Excel 2017 (Social Survey Research Information Co., Ltd.; Tokyo, Japan).

## Results

Table 1 lists the flexural strength and surface roughness values for the eight groups. As shown, both the flexural strength and the surface roughness were significantly influenced by the surface condition ( *p* <0.0001), with the flexural strength decreasing as a function of air-abrasion pressure. The highest flexural strength was observed for the as-sintered group. The surface roughness increased as a function of the air-abrasion pressure, and the highest roughness value was found for the 0.3AB group. Although the groups subjected to additional heat treatment showed no significant difference in roughness from the 0.2AB group, they exhibited significantly increased flexural strengths. In addition, the 0.2AB+HT1h group exhibited a comparable flexural strength to the control group.

**Table 1:** Flexural strength, surface roughness (Ra), and relative amounts of each phase for the highly translucent PSZ after air-abrasion with alumina at different air pressures and/or additional heat treatment

Group	Flexural	Surface	Relative amount of phase (wt%)		
	strength (MPa)	roughness (µm)	t-ZrO_**2**_	c-ZrO2	r-ZrO2
as-sintered	637.5 ± 72.7^a^	0.253 ± 0.045^cd^	47	53	0
control	582.7 ± 33.5^ab^	0.095 ± 0.010^f^	45	50	5
0.1AB	431.6 ± 35.1^cd^	0.175 ± 0.029^e^	45	50	5
0.15AB	376.5 ± 22.2^de^	0.200 ± 0.023^de^	44	50	6
0.2AB	352.6 ± 19.6^e^	0.309 ± 0.023^bc^	38	43	19
0.3AB	366.9 ± 26.6^e^	0.488 ± 0.045^a^	38	42	20
0.2AB+HT0	460.7 ± 44.0^c^	0.320 ± 0.011^b^	46	52	2
0.2AB+HT1h	574.6 ± 34.4^b^	0.317 ± 0.020^b^	45	51	4

Standard deviations followed by identical superscript letters indicate no significant differences in the flexural strength or surface roughness (p>0.05)

**Figure 1:**
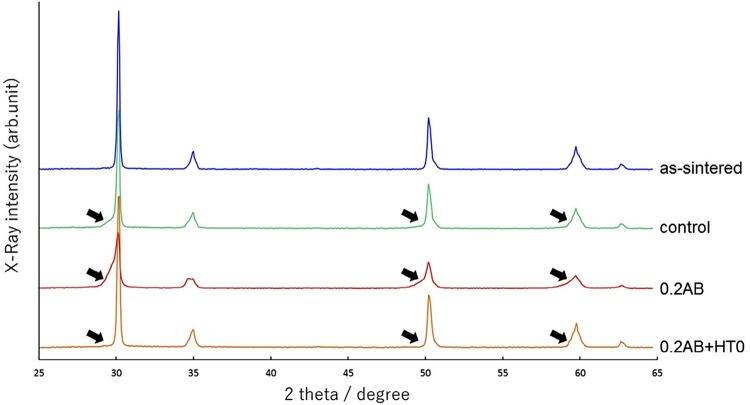
Representative XRD patterns of the as-sintered, control, 0.2AB, and 0.2AB+HT0 groups for the highly translucent Y-PSZ. Three peaks at around 29.7°, 49.5°, and 58.7° 2θ (arrows) implies the presence of r-ZrO2 phase was significantly observed for the 0.2AB group

Representative XRD patterns of the as-sintered, control, 0.2AB, and 0.2AB+HT0 groups are shown in Figure 1 . The relative amounts of each phase were listed in Table 1 by Rietveld analysis. All group surfaces contained the *c* -ZrO_2_, *t* -ZrO_2_, and *r* -ZrO_2_ phases, with the exception of the as-sintered group. Upon increasing the air-abrasion pressure, the relative amount of *r* -ZrO_2_ phase increased, with a significant amount of *r* -ZrO_2_ phase (around 20 wt%) being detected for the 0.2AB and 0.3AB groups. Following additional heat treatment, the amount of the *r* -ZrO_2_ phase decreased significantly, and the amount of the *t* -ZrO_2_ phase was comparable to the as-sintered and control groups.

**Figure 2:**
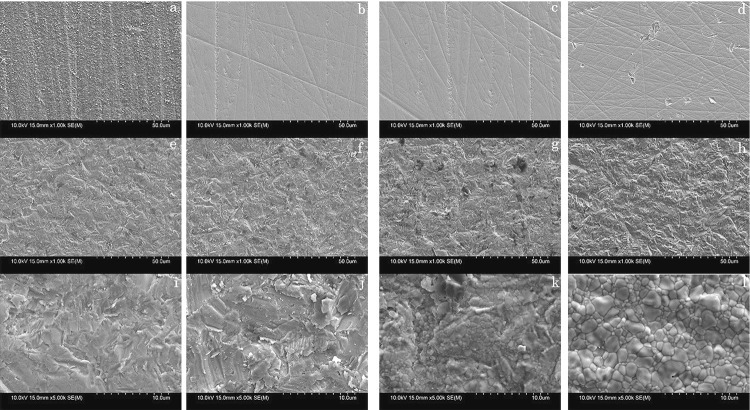
SEM images of highly translucent Y-PSZ after air-abrasion with alumina at different air pressures and/or with additional heat treatment: (a) as-sintered (1000×), (b) control (1000×), (c) 0.1AB (1000×), (d) 0.15AB (1000×), (e) 0.2AB (1000×), (f) 0.3 AB (1000×), (g) 0.2AB+HT0 (1000×), (h) 0.2AB+HT1h (1000×), (i) 0.2AB (5000×), (j) 0.3AB (5000×), (k) 0.2AB+HT0 (5000×), (l) 0.2AB+1h (5000×)

SEM images of all groups are shown in Figure 2 where it is apparent that the surface of the control group exhibited various superficial flaws such as scratches, while the 0.1AB and 0.15AB groups exhibited only a small number of defects. In contrast, numerous micro irregularities formed on the surfaces of the 0.2AB and 0.3AB groups, and the microstructures of the two heat-treated groups were very similar to that of the 0.2AB group at a magnification of 1000×. However, at a magnification of 5000×, the surface of the 0.2AB+HT0 group exhibited a degree of crystal precipitation, while that of the 0.2AB+HT1h group showed clear crystal grain boundaries.

The tensile bond strengths of the various test groups are shown in Table 2 . As indicated, the 0.2AB groups of both TZP and PSZ showed significantly higher tensile bond strengths than the control groups at TCs of 0 and 20,000 ( *p* <0.05). In addition, the 0.2AB+HT1h group exhibited a significantly lower bond strength than the 0.2AB group ( *p* <0.05), in addition to a similar bond strength to the control group ( *p* >0.05). There is no significant difference in bond strength between before and after TCs only for the 0.2AB/TZP group ( *p* >0.05). All specimens across the 0.2AB groups for both TZP and PSZ revealed cohesive failure at TCs of 0 and 20,000. In contrast, the other three groups exhibited mixed failure at TC0 and adhesive failure at TC20000.

**Table 2:** Tensile bond strength of resin cement to highly translucent TZP and PSZ after air-abrasion with alumina and/or additional heat treatment

Group	Tensile bond strength (MPa)	
	TC0	TC20000
TZP/control	25.8 ± 2.6^b,A^	9.4 ± 1.1^c,B^
TZP/0.2AB	37.8 ± 3.1^a,A^	34.5 ± 3.1^a,A^
PSZ/control	20.0 ± 2.1^c,A^	9.1 ± 1.8^c,B^
PSZ/0.2AB	38.9 ± 3.7^a,A^	29.2 ± 2.5^b,B^
PSZ/0.2AB+HT1h	20.3 ± 1.5^c,A^	8.1 ± 0.8^c,B^

Standard deviations followed by identical superscript lowercase letters indicate no significant differences in the group at same TCs (p>0.05). Standard deviations followed by identical superscript uppercase letters indicate no significant differences in the TC at same groups (p>0 .05).

## Discussion

It is widely known that micromechanical retention is necessary to enhance the bond strength of resin cement to conventional Y-TZP.^23 , 24^ Several studies^25 - 28^ have evaluated the effect of the surface treatment of conventional Y-TZP on the mechanical properties. However, few studies have considered the influence of alumina air-abrasion on the flexural strength or surface characteristics including crystallographic phase of highly translucent Y-PSZ,^21 , 22^ or on the bond strength of resin cement to Y-PSZ. In the present study, air-abrasion with alumina at different air pressures and/or with additional heat treatments were found to influence the flexural strength and surface characteristics including the crystalline phase composition, although the bond strength of resin cement to highly translucent Y-PSZ was not influenced, and so the null hypothesis was partially rejected.

The standard for testing the strength of ceramics has been the 3-point flexural test.^29^ However, this test is highly dependent on the superficial finish of the edges of specimens.^30^ It is necessary to evaluate and compare the strength between sample groups that are only as-sintered without any procedures and treatment and those that are treated with air-abrasion and/or heat treatment. Chipping and fractures may occur when performing edge chamfer of specimens. For these reasons, in this study, edge chamfer was not performed. As fracture begins at the edges, the strength values show large variation with the 3-point flexural test.^15^ On the contrary, the biaxial flexural test is recognized as a reliable method, as edge failures are eliminated because they are not directly subjected to the load,^31^ thereby producing less variation in the strength values.^15^ Coefficients of variation in the biaxial flexural tests were at most 20.3%^15^ or 10.6%,^29^ respectively, while the corresponding values from the 3-point flexural test in this study were up to 11.4%. Although fracture initiation of the specimens was not confirmed at the flaws, the 3-point flexural test was performed in this study because the specimens could be prepared finely and easily using high CAD/CAM technology.

As the air-abrasion pressures in the conventional Y-TZP and highly translucent Y-PSZ were 0.25 MPa^6^ and 0.1 MPa^32^ or 0.2 MPa,^22 , 33^ respectively, the range of pressure in air-abrasion was 0.1 to 0.3 MPa in this study. Air-abrasion with alumina also caused a negative impact on the conventional Y-TZP,^17^ as this abrasion reduced the strength of Y-TZP due to the appearance of micro-cracks on the surface, which can lead to failure. In contrast, a positive impact has also been reported due to phase transformation from the *t* -ZrO_2_ phase to the *m* -ZrO_2_ phase.^15 , 25 , 27^ Upon abrasion of the conventional Y-TZP surface with alumina, the *t→m* phase transformation occurs, resulting in a 3-5% local increase in volume, which promotes the concentration of compressive stress around superficial defects and increases the fracture toughness.^34^ Thus, the balance between the flaws and the compressive strength created by the *t→m* phase transformation on the surface layer can ultimately define the positive or negative impact on the mechanical properties. If the flaws are confined inside this layer, a positive impact is observed,^34 , 35^ while a great number of flaws that overcome any increase in the compressive stress may negatively impact the mechanical properties.^36^

In the present study, upon increasing the pressure of alumina air-abrasion, the flexural strength of the highly translucent Y-PSZ decreased, which is a similar result to previous studies.^21 , 33^ These studies also reported that the decrease in the flexural strength of Y-PSZ after alumina air-abrasion was due to the reduced *t→m* phase transformation in the presence of a high *c* -ZrO_2_ phase content.^21^ After grinding the Y-PSZ specimens with SiC paper, each diffraction line was broadened, and some new broadened diffraction lines were observed at the lower angles of the diffraction lines corresponding to the *c* -ZrO_2_ phase. These observations suggested the formation of a new phase due to the transformation of the *c* -ZrO_2_ phase under stress, and was identified as the *r* -ZrO_2_ phase,^37^ which is also formed from both the *c* - and *t* -ZrO_2_ phases in fully and partially stabilized zirconia.^38^ In contrast, a broadened *t* -ZrO_2_ peak was observed for the conventional Y-TZP^39 , 40^ and for the highly translucent Y-PSZ^32^ after grinding or air-abrasion with alumina. In this case, this was assumed to originate from the *r* -ZrO_2_ phase because three peaks at around 29.7°, 49.5°, and 58.7° 2θ were observed in agreement with the previous study.^22^ The relative amounts of each phase were also calculated by Rietveld refinement. Thus, it was found that the presence of around 20 wt% of *r* -ZrO_2_ phase and no *m* -ZrO_2_ phase for the highly translucent Y-PSZ specimens subjected to alumina air-abrasion at 0.2 and 0.3 MPa. In addition, no microcracks were observed on the air-abraded surface of the highly translucent Y-PSZ, and so the decrease in the flexural strength of this material following air-abrasion with alumina was attributed to the increased *c→r* phase transformation.

As numerous micro irregularities observed on the surfaces of highly translucent Y-PSZ air-abraded with alumina from 0.2 MPa by SEM to obtain micromechanical retention of resin cement, the adequate pressure for Y-PSZ was decided at 0.2 MPa. Therefore, in the present study, additional heat treatment was performed only for the 0.2AB group. Following additional heat treatment was performed only for the 0.2AB group. Following additional heat treatment, the flexural strength was recovered to a level similar to the control group. Furthermore, the relative phase composition of *t* -, *c* -, and *r* -ZrO_2_ phases for the 0.2AB+HT1h group was comparable to that of the control group. With the progression of the *c→r* phase transformation upon air-abrasion of the highly translucent Y-PSZ, strain accumulates in the surface layer of the bulk material. It was therefore suggested that upon additional heat treatment, the residual stress was released, and as a result, the *r→c* reverse phase transformation occurred. Furthermore, the surface images of the air-abraded highly translucent Y-TZP and Y-PSZ specimens were comparable,^12 , 19^ thereby confirming that the difference in behavior of flexural strength between these two materials may be due to a lack of transformation toughening in the case of the highly translucent Y-PSZ.

Monolithic zirconia crowns are commonly luted on substrates fabricated by foundation resin composite with resin cement. Therefore, most studies have prepared zirconia specimens bonding resin composite with resin cement.^7 - 9 , 11 , 32^ However, some specimens were fractured within the resin composite, due to which the true bond strength of the resin cement to zirconia could not be evaluated. Resin cement containing an adhesive phosphate monomer such as MDP, which is also a component of the G-CEM ONE used in this study, bonded strongly to stainless steel (SUS 304) and showed durable bond strength.^41^ Therefore, in this study, zirconia was bonded to a stainless steel rod instead of a resin composite with resin cement.

It is important to compare the influence of alumina air-abrasion on the bond strength of resin cement between highly translucent Y-PSZ and Y-TZP. As additional heat treatment is unnecessary for the air-abraded TZP, it was performed only for the PSZ specimens. Although additional heat treatment resulted in recovery of the flexural strength and surface characteristics of the highly translucent Y-PSZ air-abraded at 0.2 MPa to the level of the control group, the group subjected to additional heat treatment exhibited a significantly lower bond strength than the group only subjected to air-abrasion. More specifically, alumina air-abrasion left a roughened surface with expected higher wettability,^42^ and its significant reduction after heat treatment may lead to a decreased bond strength between resin cement and the Y-PSZ. In contrast, air-abrasion at 0.2 MPa did not affect the bond strength of resin cement although the flexural strength decreased and the crystallographic phases present were altered compared with the control group. Note that in the case of conventional Y-TZP air-abraded with 110-µm alumina at 0.4 MPa for 15 s, *t→m* phase transformed zone depth is approximately 0.3 µm,^43^ which is much lower than the cement thickness (50 µm) in this study. Thus, the influence of decreased flexural strength of highly translucent Y-PSZ on the bond strength of resin cement would be negligible after alumina air-abrasion.

From the obtained results, it is apparent that micromechanical retention by air-abrasion with alumina is essential to obtain durable bonding of resin cement to highly translucent Y-PSZ and Y-TZP. Different air-abrasion conditions affected the bond strength of resin cement in the case of the highly translucent Y-TZP air-abraded with between 50-µm alumina at 0.2 MPa and 30-µm alumina at 0.12 MPa.^44^ Water storage for 3 months resulted in the reduction of microtensile bond strength for the group air-abraded with smaller alumina particles and lower pressure. When alumina air-abrasion was used to treat the inner surface of zirconia crowns, even with larger particles, the system behaved as a bonded crown, promoting a higher fatigue resistance for the cemented crowns.^45^

## Conclusions

This study has shown that the flexural strength of highly translucent Y-PSZ decreased with the increase in air-abrasion pressure with alumina. The lowest flexural strength and the highest roughness value were found for the 0.2AB and 0.3AB groups, respectively. Upon increasing the air-abrasion pressure, the relative amount of the rhombohedral ( *r* )-ZrO_2_ phase increased, with a significant amount of *r* -ZrO_2_ phase being detected for the 0.2AB and 0.3AB groups. The 0.2AB+HT1h group exhibited a similar flexural strength and tetragonal-ZrO_2_ phase content as the as-sintered group. However, the 0.2AB group showed a significantly higher tensile bond strength than the 0.2AB+HT1h group before and after aging. These findings suggest that micromechanical retention by alumina air-abrasion, in combination with chemical bonding of a resin to highly translucent Y-PSZ using a MDP-containing resin cement may enable durable bonding, which is similar to the conventional and highly translucent Y-TZP.
